# The policy construction of illegal markets: Exploring variation, success, failure, and unintended consequences

**DOI:** 10.1073/pnas.2515300123

**Published:** 2026-08-10

**Authors:** Letizia Paoli, Peter Reuter

**Affiliations:** ^a^ Department of Criminal Law and Criminology, Faculty of Law and Criminology, KU Leuven, Leuven 3000, Belgium; ^b^ School of Public Policy, University of Maryland, College Park, MD 20742; ^c^ Department of Criminology, University of Maryland, College Park, MD 20742

**Keywords:** illegal markets, regulation, prohibition, harm reduction, drug policy

## Abstract

Illegal markets, broadly defined to include markets where only specific transactions are illegal, are the consequence of policy, either a prohibition or highly restrictive regulation. They are generated by a variety of concerns, some moral, some pragmatic. They are often tied to legal markets, complicating the policy process. These markets are ubiquitous, rarely well controlled and little studied as markets. This article examines variations in illegality from the case studies in this Special Feature and synthesizes what can be learned about the success and failure of different approaches to controlling these markets. Control is often weak because powerful legal interests are opposed to tight regulation and, especially in markets with large underlying demand, rarely leads to substantial reductions in the targeted activity, unless draconian methods are used. As demonstrated by drug markets, tough enforcement can have large adverse collateral consequences. The paper concludes by suggesting that an extension of the harm reduction framework developed specifically for drug policy can be applied usefully to making policy choices in controlling other illegal markets.

Illegal markets, broadly defined to include markets where only some specific transactions are illegal, are ubiquitous, though unevenly distributed. Almost every nation has a substantial market for counterfeit goods and an illegal drug market. Many nations also have a market in tax-evaded cigarettes and prohibited commercial sex. A few have a substantial trade in protected wildlife; a different few import many stolen antiquities. Some are the source of individuals who want to enter a country that does not permit them entry; a smaller number of countries defend against human smugglers and their customers. A major goal of this essay is to draw on the individual market studies in this *PNAS* special issue and on other studies to assess what in fact might be learned by concentrating on the commonality of being an illegal market.

Each illegal market can be regarded as an unintended byproduct of the growing government regulation of business and other social activities. Such regulation has progressively complemented and partially offset self-regulation and, in Europe, public ownership, intensifying significantly from the mid-19th century onward (1, 2). More specifically, most illegal markets since World War II reflect the growing reliance on criminal law in regulatory efforts to ban or restrict goods, services, or specific modalities of production, distribution, or use that are considered harmful, immoral, and/or associated with specific “dangerous” groups (3). More recently, this trend has been compounded by the growing reliance on “quasi-criminal enforcement mechanisms” that imitate criminal law without formally carrying that label (4). In Continental Europe, these punitive enforcement mechanisms often rely on administrative procedures and sanctions, which are usually entrusted to specialized regulatory agencies. Common law systems, instead, frequently resort to civil law rules and procedures, including civil penalties, asset forfeiture and punitive damages, to achieve the same punitive aims. These trends have undermined the traditional distinction between regulation and prohibition, as criminal law is increasingly used as a regulatory tool in various combinations with other civil or administrative law measures, so that some authors, such as Bailleux (5), have spoken of “criministrative law.”

On the wave of multifaceted and often idiosyncratic regulatory efforts and given a persistent or rising demand for the targeted products, a few wholly criminal markets have developed during the 20th century, that is, markets where all activities, both on the demand and on the supply side, are prohibited by criminal law. At the same time, the number of manufacturing and trading activities subjected to restrictions via criminal or other types of law (e.g., health and safety regulations) has multiplied, inadvertently leading to the rise of what Paoli and Lord (6) have called “variably legal markets.” In these markets, some activities are criminalized or restricted with either criminal or otherwise punitive, “quasi-criminal” enforcement tools and mechanisms of administrative, civil, or even hybrid nature (7), while other activities are not. In “variably legal markets,” the legal status of the exchanges and/or their parties is conditional and fluctuating across time and space.

We provide many instances of such markets in the following paragraph, but we first discuss the case of illegal drug markets, which constitute the clearest example of wholly criminal markets. Drugs that are now prohibited were perfectly legal in most countries until the early 20th century. Famously, the British fought two “Opium Wars” with China (1839–1842 and 1856–1858) to open up that nation to opium exports from India (8). Cannabis, the most popular plant-based illegal drug, was cultivated—and also grew wildly—in at least four continents and until the 1920s “the large majority of countries did not have any controls at all” (9). However, the 1961 UN Single Convention on Narcotics Drugs virtually prohibited all production and distribution of many prominent psychoactive substances, including heroin, cocaine, and cannabis; it also prohibited possession. Since then, drug transactions have grown to form what is generally believed to be the largest illegal market globally. For the United States (US), an estimate for 2016, the latest available, was over $150 billion (10).

Next to these and a few other wholly criminal markets, thousands of illegal exchanges occur daily involving a multiplicity of restricted, but not completely prohibited, goods and services—from firearms to fish, from gambling to sex services—as part of broader “variably legal markets” (7). Illegal fishing, for example, is largely carried out by the same vessels that account for most legal fishing. The vessels’ activities become illegal when fishing occurs within waters under the jurisdiction of a state or Regional Fisheries Management Organization (RFMO) in violation of the latter’s regulations (11). More recently, the partial decriminalization and legalization of some activities and/or products within established criminal markets have also created further variably legal markets, as in the case of recreational cannabis (12) and gambling (13).

Against this background, this article examines and compares the regulatory frameworks and control policies of a variety of wholly illegal and variably legal markets, aiming to identify commonalities and differences that might help improve such regulation and policies. It focuses on the markets that are analyzed in the nine chapters within this Special Feature, although we occasionally also refer to other illegal markets and exchanges. Because of our focus on policies, our analysis is distinct from the parallel scientific synthesis chapter of Caulkins (14).

The article is organized as follows. Section 1 compares the motivations for the bans that have led to the creation of the selected illegal markets and exchanges. Section 2 examines variations of illegality across the selected markets in terms of the extent of the bans, the types of laws applied and the sanctions imposed. Section 3 examines the variable relation of illegal markets to their legal counterparts and the mingling of illegal and legal exchanges. Section 4 assesses variations in enforcement. Section 5 reviews what is known about the success, failures, and unintended consequences of control efforts. The final section sums up the main findings, reviews the principles discussed in the literature to justify criminalization and public enforcement tools and, on this basis, calls for the development of “mixed” policy strategies inspired by the principle of harm reduction.

## 1. Motivations for Banning

The motivations for the bans have varied. In most cases, the bans have been meant to serve the consequentialist goal of protecting particular legal interests. The interests of the general population have often been prioritized: for example, health, in the case of illegal drugs, or safety, in the case of illegal firearms. Such broad bans usually focus not only on harm to others, the proper and exclusive aim of criminal prohibition according to most economists and some other scholars (15, 16). Drug prohibitions, especially those extending to buyers’ possession and personal use, have also been justified by the failures of self-control (17). Prohibitions have also been issued to protect children and other vulnerable individuals from being exploited in the sex or other markets. In virtually all jurisdictions, for example, prostitution of minors and forced prostitution are prohibited.

In other instances, the bans are primarily intended to protect the legal interests of specific collective stakeholders. The prohibition of product counterfeiting, for example, primarily serves to protect businesses’ intellectual property. Since the consolidation of nation states in the 17th century, restrictive rules have also often been issued to protect state interests (2), including tax revenues, as in the case of the ban on smuggled cigarettes, or the national borders, as in the case of human smuggling. The prohibition on trade in certain wildlife (18) and the growing restrictions on fishing (11) reflect the fact that animals are increasingly considered worthy of legal protection for themselves and not just as means for fostering human welfare.

The protection of multiple—and sometimes conflicting—interests often inspires a ban. Fishing, for example, has been under certain conditions prohibited to ensure the sustainability of fish populations and also to guarantee long-term food security, fisheries, and the livelihood of regions heavily reliant on fishing. The protection of human health and of the financial interests of tobacco manufacturers constitute further justifications for the ban on illegally manufactured cigarettes in addition to the protection of tax revenues. The relative importance of the different legal interests deemed worthy of protection varies across time and jurisdiction. Gambling offers a clear example. In the US, almost all forms were prohibited until 1970. In 2025 the legal gambling industry generates tens of billions of dollars annually, takes many forms (lotteries, casinos, etc.) and is a major source of state revenues (19). At the same time, China prohibits all forms of gambling, except the state-run lottery.

In the bans on prostitution and/or its supporting activities such as public soliciting, procuring, and the operation of brothels, consequentialist goals have mostly been subordinated to moral considerations. The vision underlying total bans, such as those in force in most US states, is that commercial sex constitutes a repugnant activity (20). A similar view also drives the “abolitionist” policies that have been adopted in many European countries since WWII. These allow prostitution, but prohibit many of its supporting activities, including soliciting in the “neoabolitionist” model of Sweden and other countries, where the customers, but not the prostitutes themselves, are subject to criminal sanctions. An additional assumption of both variants of the abolitionist approach is that prostitutes, assumed to be all female, are victims of criminal—and male—exploitation and hence need protection (21).

Realpolitik—or business—considerations have also occasionally motivated ban campaigns. The early US initiatives to ban opium, for example, were dictated not only by moral concerns but also by political and economic interests. The US was eager to end the profitable opium trade dominated by Britain and other colonial powers and to curry favor with the Chinese, thereby improving Sino-American economic relations (22). Well into this century, many international initiatives meant to control the spread of counterfeit medicines were spearheaded by pharmaceutical companies eager to protect their brands and to target legitimate generic medicines under the guise of IP enforcement (23). Recognizing the confusion caused by the term “counterfeit,” in 2017 the WHO has moved away from using it in favor of terms like “substandard” and “falsified” to emphasize that its primary concern is patient harm, not patent status (24).

Last but not least, chance and epochal events have sometimes had a lasting impact on bans and other restrictive regulation. In 1919, for example, the Supreme Court upheld a prohibitionist interpretation of the Harrison Act of 1914, the first federal drug control law, despite the Act’s original intent to “medicalize” opium and cocaine, in a 5 to 4 decision (22, 25). Given the opposition of Germany, the first international drug control treaty, the International Opium Convention of 1912, might never have entered into force had the British government not made its ratification a condition of the Treaty of Versailles that ended World War I in 1919 (26). At least as far as drugs are concerned, historical research “demonstrates that the concepts, the reactions, the structures of controls which are now taken for granted are not fixed and immutable” (27).

## 2. Variations in Illegality

Illegal markets are characterized by variations of illegality in terms of the extent of the bans, the types of laws applied, and the sanctions imposed.

Consider first the extent of the bans. In some markets and places, both the sales and purchases are criminalized. All suppliers as well as the buyers of drugs controlled under Schedule I of the Single Convention commit criminal offenses according to this and related UN conventions and the national legislations that literally implement these international treaties (for deviations, see below). About half the countries in the European Union specify even drug use or consumption as a criminal offense (28). Similarly, both prostitution and the solicitation for prostitution constitute criminal violations in most US states (20).

Such sweeping criminalization is not always the norm. In some illegal markets, only the buyers are targeted. In neoabolitionist—or End Demand—prostitution regimes, implemented in Sweden, France (29) and, since 2023, ME (30), the buyers of sex services (along with pimps and other presumed exploiters) commit criminal offenses, whereas the prostitutes are not subject to criminal sanctions. In the US regulatory framework for firearms, the goal is to prevent people who have a serious criminal record, and a few other categories of individuals, from acquiring a firearm. Such persons hence commit a criminal offense if they try to do so. Federally licensed retail dealers are only required to check that their customers satisfy state and federal requirements and keep a record of each sale. Individuals selling or otherwise transferring their firearms on the secondary informal market are even exempted from such screening and recordkeeping requirements (31).

More often, the burden of prohibition largely falls on suppliers. Trafficking in counterfeit goods, for example, is criminalized in most jurisdictions. Conversely, the purchase of goods bearing counterfeit marks is not a crime in the US, if it is done for personal use (32). Likewise, the UN Protocol against Smuggling of Migrants (33) requires countries to criminalize smugglers, but not the migrants themselves. Until recently, the dominant idea was, in fact, that migrants should not be criminalized solely for being smuggled, especially if they are seeking asylum or are victims of exploitation, though the legislation has become more severe in many contexts (34).

One factor explaining the differential criminal liability is that the final customers might not even know that they are buying an illegal good. This is, for example, often the case of the research centers importing macaques for medical purposes. Although such centers receive assurances that the macaques were captive bred, these monkeys are often poached and falsely labeled in breach of the Convention on International Trade in Endangered Species of Wild Fauna and Flora (CITES) (35) and the attendant national legislations in both the exporting and the importing countries (36). *Mutatis mutandis*, the same can be said of illegal fishing. End-customers and even many intermediary businesses along the supply-chain are rarely aware that the fish they are buying was illegally caught, as the illegal catch is sold alongside the legal catch (11).

In some illegal markets, moreover, the final buyers are not perpetrators, but at least potentially victims. Among the latter are the buyers of some counterfeit goods, especially medicines, many of which are substandard and circulate widely in many countries of the Global South. In 2017, the World Health Organization (37) estimated that 1 in 10 medical products in low- and middle-income countries is substandard or falsified.

Some illegal markets exist only in specific jurisdictions. The differences across countries are most pronounced in the case of prostitution. While prostitution and soliciting are banned in most US states, Russia, China, and many other Asian states, both are legal under certain conditions in Germany, the Netherlands, and Switzerland (21). Similarly, sex work is largely treated as a legitimate occupation, subject to standard labor laws in New Zealand and Belgium (38). Even in these jurisdictions, though, some forms of prostitution are not allowed. Street prostitution, for example, has been administratively prohibited by most Dutch cities (39), while sex workers active in Germany are required to register. Those who do not do so—because they do not want to be classified as prostitutes or to pay taxes, or because they do not have an address or legal residency status in Germany—are breaking the law (40). Hence prostitution in these countries is a mix of legal and illegal activities; the latter mostly entail breaches of administrative or tax laws, but some criminal prohibitions still remain, for example, those targeting underage or forced prostitution.

Since 2000, legal markets have also started to re-emerge for recreational cannabis, the most frequently used illegal drug worldwide (41). As of May 2025, 24 US states have—with some differences—legalized the production, distribution, and purchase of cannabis for recreational use, even though all these acts remain prohibited by the UN Conventions and by US federal law. Other jurisdictions—e.g., Canada and Uruguay in the Western hemisphere as well as Luxembourg, Malta, and Germany in Europe (41)—have followed through, albeit much less sweepingly than US states.

There are also differences in the types of laws applied and the sanctions imposed. For drugs, they both vary by the nature of market participation. Individuals who use illegal drugs, at least in Europe, are less and less subject to criminal penalties and often only subjected to administrative sanctions—or sometimes no sanction at all, even if they are guilty of a criminal offense (42). Suppliers of illegal drugs, though—particularly if they operate at the wholesale level and/or are considered members of organized crime groups—are virtually everywhere pursued with criminal law. If convicted, they are routinely given lengthy prison sentences (43)—and, in some non-European countries, even death (44). At least occasionally, the suppliers of contraband cigarettes, illegal wildlife, and (more often) human smuggling services have also undergone criminal trials and received heavy penalties, including lengthy prison sentences.

Corporations or their legal representatives, however, are rarely charged—and even more rarely convicted—of criminal offenses, and few, if any, of their representatives have ever gone to prison. Since the early 21st century, for example, most major banks in both the US and Europe—such as Wachovia Bank in the US and, more recently, HSBC, Danske Bank, and Deutsche Bank in Europe—were found to have facilitated money laundering for Mexican drug trafficking organizations, “shady” clients in Russia and other former Soviet states or other individuals and businesses subject to US sanctions (45). The total amounts laundered in each bank were considerable, ranging from hundreds of millions of US dollars to more than $200 billion in the case of the Estonian branch of Danske Bank. Yet, criminal law has so far been used sparingly and combined with both civil and administrative sanctions. Obviously, there is no comparable punishment to imprisonment for corporations; nonetheless, the choices of the courts and other regulators feed the longstanding supposition of many scholars and other observers that white-collar offenders get a preferential treatment vis-à-vis ordinary criminals (46).

## 3. Illegal Markets in the Shadow of Legal Markets

Illegal markets differ not only in their legal framework but also in their relation to the corresponding legal markets. In only a few cases is there virtually no parallel legal market. The legal production of heroin, for example, only occurs in a few countries and serves heroin maintenance clinics and/or the national health services. Legally manufactured heroin cannot be bought by individuals or businesses (6). In other words, there are only rare legal exchanges involving heroin; they are dwarfed by the illegal market. With few exceptions, there is hardly any longer a legal ivory trade. After CITES banned international trade in ivory from African elephants in 1989, the legal trade in ivory has progressively diminished. Only limited trade in certified antiques is still allowed (47). All the remaining trade in ivory is illegal.

At the opposite end of the spectrum, many other illegal markets are closely tied to their legal counterparts. Indeed, they may not even constitute separate markets and the illegal exchanges in the banned products constitute part of broader variably legal markets (6). Money laundering is simply the process by which criminal revenues are brought into legal finance. Fish and other types of wildlife, once caught or poached, are also traded along the same routes as legal wildlife. The legal ambiguities are in this context so significant that illegal fishing is studied and tackled together with unreported and unregulated fishing (hence the standard acronym IUU). (18).

Whereas wildlife, and presumably other natural resources are caught/extracted illegally and then mingle with their legal counterparts, the opposite trajectory can be identified for many counterfeit products. Kenkel and Philips (48), for example, note that “most cigarettes sold in illegal markets are manufactured legally.” Other counterfeit goods, such as handbags and other apparel, are also produced legally and become illegal only when the logo of a specific brand is applied on them.

That many illegal markets—or exchanges—are interwoven with their legal counterparts implies that it is necessary to intervene in the latter in order to contain the former. The principal instrument of money laundering controls is the requirement that financial institutions report “suspicious activities,” often involving attempts to disguise the criminal origins of the funds (49). IUU fishing is contained in large part by monitoring the activities of large legal trawlers at sea to prevent mingling of lawful catch with unlawful catch. Restricting some kinds of counterfeit goods (notably high-quality counterfeits) also requires monitoring of legal retailers who may sell both legal and counterfeit products.

To pick up on an earlier theme, the mingling of legal and illegal commerce creates dilemmas for policymakers, who need to decide which interests to recognize as worthy of legal protection and how to weigh competing interests. By prohibiting the catch and sale of fish or other wildlife species, policymakers intend to protect the sustainability of the animals and the long-term sustainability of the ecosystems. At the same time, though, they hurt—at least in the short term—the financial interests of several large and small businesses, including subsistence fisheries that might have difficulties finding an alternative livelihood. Another example concerns the generic drugs produced in India or other developing countries without complying with the patents of western pharmaceutical companies. The latter often refer to these generic drugs as “counterfeit,” try to stop their production and undoubtedly see reduced returns on their investments. However, it is well established that Indian manufacturers have saved millions of lives by producing low-cost antiretroviral drugs and other generics in compliance with their domestic legislation and an expansive interpretation of the so-called TRIPS agreement of the World Trade Organization (50). The key question hence becomes whose legal interests ought to be prioritized.

## 4. The Vagaries of Enforcement

Possibly reflecting the conflicting interests to be balanced, in many illegal markets there is a considerable difference between the “law on the books” and the “law in action” or between “formal” and “substantive criminalization”(51). Law enforcement—broadly understood—is, in other words, not consistent and often weak. By weak enforcement, we mean that the risk of apprehension is low and that penalties or other sanctions, conditional on being caught, are not large enough to serve as a deterrent.

Examples of weak enforcement abound in the markets of this Special Feature. For example, many countries with strong money laundering control legislation, as required by the Financial Action Task Force, make minimal enforcement efforts (52). Even in the US, the nation most responsible for the creation of the existing control system, there are barely 1,000 convictions annually, whereas it is likely that hundreds of billions of dollars are laundered each year (45). Though there are no estimates of the number of individuals who launder significant quantities, it is surely in the many tens of thousands. Likewise, the formal prohibition of the purchase of counterfeit goods and even cigarettes for personal use is hardly ever enforced, even in the countries where it constitutes a criminal offense. Weak enforcement also often extends to the supply. A 2015 National Research Council report on illegal cigarette markets in the US concluded that “law enforcement efforts attempting to detect and investigate the illicit trade have been weak and uneven and even when inspections are increased, the number of packs seized has fluctuated around very low levels” (53).

For some markets, enforcement is just nominal. Midgette and Gore (18) report that the principal international enforcement agency for international wildlife trafficking has a total annual budget of $14 to 20 million to control a trade that crosses many borders. Countries that are the source for most species of trafficked wildlife are poor, with limited governance capacity. In other cases, sanctions are simply too low to make a difference. Sumalia (54) estimates that even with a 20% detection rate, far above the estimated current figure, penalties would have to rise 24-fold to make IUU fishing unprofitable. Moreover, when vessels and crews are detected in IUU activities the owners can often avoid criminal penalties through complex ownership structures.

Only perhaps for some illegal drugs in a few countries, such as the US (55) or Singapore (56), is the level of enforcement rigorous. Even for illegal drugs, though, the picture is quite varied. In addition to the legal changes discussed in the previous section, some countries have decided to stop enforcing some provisions of their drug law. Even before the formal legalization of recreational cannabis, many US states allowed marijuana dispensaries—supposedly just for patients whose doctors had recommended cannabis for therapeutic purposes—to supply recreational users, as well (9). Although the Dutch drug legislation remains very strict on paper (28), Dutch authorities have since the mid-1970s allowed limited retail sales of cannabis for recreational use via the so-called “coffee-shops.” This tolerance policy has led since the 1990s to a de facto legalization of certain parts of the cannabis supply chain (57), though it has not addressed the supply of the coffee-shops which are forced to buy cannabis on the illegal market [the-so-called “back door problem;” see (58)].

Because of these enforcement inconsistencies and weaknesses, the question is often not “why is the market so big?” but rather “why isn’t the market bigger?” since the activity seems low risk and highly profitable. Indeed, the 2015 National Research Council Report on the illegal cigarette market in the US (53) concluded just that: “Given the low risks and high profits…of the illicit tobacco trade, the obvious question is why the illicit market in the US is not larger.” (p.155). While we do not have the space to articulate a full answer to that question, we note that cultural factors and specifically moral norms certainly play a role. The widespread gun ownership in the US has been linked to a distinctive gun culture shaped by frontier experiences, civilian militias, and, as Haag (59) argues, the strategic efforts of gun manufacturers at the turn of the twentieth century. In Europe, instead, most people are not interested in owning a firearm, as EU surveys show (60).

Cultural and socioeconomic factors also explain why the enforcement of many bans is inconsistent and weak even in many countries with a strong state. In democratic societies committed to the rule of law, the enforcement of many bans—even of drug laws, usually the most stringently applied—is constrained by the obligation to respect the human rights of the users and suppliers.

More generally, as noted above, both regulators and enforcers have to balance competing interests. Even in the case of drugs, policymakers and enforcers pay at least some attention to the financial interests of the businesses that inadvertently facilitate the drug trade. At the Antwerp port, which has become a major entry point for cocaine for whole of Europe, less than 2.5% of all containers were checked as of 2021 (61). Despite their growing concern for drug smuggling and the associated corruption and violence, neither port regulators nor the logistic companies—in Antwerp and elsewhere—want to further slow down the handling of containers, as their primary aim is to remain competitive vis-à-vis other ports.

Control efforts are also weakened by the persistent and apparently irreducible large demand for many illegal/restricted goods and services. Kennedy (62), for example, has reached the following, pessimistic conclusions for counterfeit goods: “While brand owners, law enforcement agencies and e-commerce platforms have taken substantial steps forward in their anticounterfeiting efforts, these activities appear to have had little impact on the primary driver of counterfeiting: consumer demand.” A strong demand also hampers efforts to reduce the size of other markets, including those for contraband cigarettes, illegal drugs, commercial sex, and smuggling services. For example, Kilmer (12) estimates that there are between two and five billion purchases of illegal drugs each year in the US.

Given this persistent demand, transactions are consensual, which implies that policing requires active investigation. No one is likely to complain about money being laundered or cigarettes being sold without appropriate taxes being paid. Indoor prostitution attracts few complainants. Policing without complainants is notoriously difficult (63).

The control of many of these markets also requires international cooperation for criminal cases. For example, money laundering frequently crosses multiple national boundaries; the laundering of the proceeds of fraud in Spain might involve a Liechtenstein trust and a British Virgin Islands shell company. Investigation is then made much more complex in legal terms. The same is generally true of International Wildlife Trafficking, IUU, and important elements of the illegal cigarette market. Small countries with relatively biddable public officials can provide gaps in coverage of international agreements. Le Billon et al (11) note that phenomenon for IUU controls. A number of small jurisdictions such as the Cayman Islands, Liechtenstein, and Jersey have played an important role in the laundering of money (64).

## 5. Successes, Failures, and Unintended Consequences of Control Efforts

Despite the multiple challenges, the policy picture is not totally negative; there are a few success stories. Some illegal markets have been reduced in size, mostly due to interventions other than strict law enforcement alone. Payments by EU to transit countries, notably Türkiye in 2016, to keep refugees and asylum seekers led to large reductions in the flow of illegal immigrants (mostly from the Middle East) to Europe through the Eastern Mediterranean route (65). The flow of undocumented persons across the Mexican–US border was drastically reduced in 2024-2025 following a dramatic change in both US and Mexican policies. Under President Biden, the US created procedural difficulties for undocumented individuals to make asylum claims, while the Mexican government massively expanded its own programs to move those aiming to cross the border back to the interior of Mexico (66).

Several countries have also managed to improve the conservation of animals that were on the brink of extinction because of poaching or habitat loss. Since the 1970s, for example, the populations of vicuñas in the Andes (67), of tigers in India (68) and of elephants in Southern Africa (69) have rebounded considerably. In all those cases, the most effective measures included setting up high-quality protected areas and involving local communities in conservation programs. CITES listing has also had great symbolic relevance, but the enforcement of the bans—despite the establishment of wildlife crime units and specialized courts and the occasional imposition of long prison sentences in some African countries—has generally remained weak (18).

The Kimberley Process Certification Scheme (KPCS) is also—at least partially—a success story. Launched in 2003, KPCS is a coalition of governments, civil society, and the diamond industry to eliminate the trade in so-called “conflict diamonds,” that is “rough diamonds used by rebel movements or their allies to finance conflict aimed at undermining legitimate governments” (70). The KPCS relies on administrative controls to track stones from mine to export, and subsequent trading. A 2006 review concluded that, thanks to KPCS, the proportion of conflict diamonds in the global trade decreased from approximately 15% in the 1990s to less than 1% in the early 2000s (71). Independent studies also confirm—albeit do not quantify—the reduction (72), though some NGOs have pointed to KPCS’s limited effectiveness in reducing other human rights violations associated with diamond extraction (73).

For illegal drug markets, many demand-side interventions have also been shown to reduce the volume of drug use. Repeated studies, including some long-term, have reported the positive effects of drug treatment, particularly Opioid Substitution Treatment (74, 75). For some preventive interventions, especially the “social competence or life skills school-based programmes,” there is also “some evidence of positive impact over the medium to longer term” (75).

Since the 1980s, several measures have also been developed to specifically reduce the harms of drug use, rather than its incidence. There is evidence that many of them are effective, especially needle exchange programs, the distribution of Naloxone, and heroin maintenance programs (76). While no supply-side intervention has been officially flagged under such banner, police work is often directed at the reduction of the violence and public nuisance associated with drug trafficking and dealing. Zimring (77), for example, has described changes in policing in NY as a “triumph of harm reduction,” because they led to a radical decline in drug-related killings, even if drug consumption, and presumably dealing, remained stable.

Though prohibition, of itself, raises prices and reduces consumption, there is little evidence of the effectiveness of repressive measures aimed at further reducing the supply—and thus the availability and use of illegal drugs (75, 78). Though a recent review of precursor controls found that they led to price increases for methamphetamine, the effects rarely lasted for as much as one y (79). Yet, supply-reduction measures keep attracting the bulk of drug policy expenditure in most jurisdictions. According to EMCDDA data (80), more than 50% of such expenditure in France, Germany, Italy, and the United Kingdom went into supply reduction.

Up to a point, law enforcement measures are necessary and even helpful. They not only deter current and prospective offenders but have also proven to be an effective Damocles’ sword in convincing addicted suspects arrested for drug-related crime to undergo treatment instead of serving a prison sentence (81).

Yet, law enforcement measures—and particularly imprisonment—are costly and generate substantial negative unintended consequences. People imprisoned for drug offenses constitute a sizable share of the prison population in many countries, amounting to more than 340,000 individuals in the US (82) and to more than 25% of the prison population in several European countries (83, 84). Negative unintended consequences are not only due to imprisonment, but also to the prohibitionist approach itself. If prohibition is not tempered by harm reduction, in fact, injecting drug users have an incentive to share needles to avoid detection and all users are unable to check the quality of the substances they use. Suppliers resort to violence to defend their interests, as they cannot go to court.

Across many illegal and variably illegal markets, more research is necessary to understand which interventions—or combinations of interventions—are most effective and cost-effective in reducing the volume of the transactions and/or the harms associated with them. Such assessments also need to take into due account that the unintended negative consequences of an intervention of policy can be distal and subtle. There is evidence that rich countries implementing neo-abolitionist reforms have seen increased travel to destinations in the Global South known for easy access to commercial sex (e.g., Thailand and the Philippines; 20). The harms of wildlife trafficking are primarily associated with the harvesting of the wildlife, rather than the distribution or the consumption. Perhaps the most effective program for shrinking the size of the market involves interdiction close to the destination market, i.e., the reduction in consumption of, say, ivory tusks per $1 million is higher for interdiction around Singapore, a major destination market, than for $1 million spent in raising the risks faced by poachers in Africa. However, if higher interdiction rates lead to more total exports from Africa, even though a smaller quantity reaches the final market, the harms may be minimized by investing in poaching controls.

Despite the poor evidence base, it is clear that the most impressive successes in rapidly suppressing specific illegal markets have been achieved by authoritarian states that are motivated by an ideological stance overruling all other considerations. The most famous success story is that of the Chinese Communist regime’s efforts to eliminate the nation’s massive opium market (85) soon after it came to power in 1949. The Taliban as the governing authority in Afghanistan demonstrates what an effective authoritarian regime can accomplish against drug production. Since the Taliban regained power in 2021, opium production has dropped by 90% by some estimates (86), reflecting the credibility of Taliban sanctions.

Even barring such extreme cases, the comparative analysis of enforcement in multiple markets suggests a paradox. The policy struggle over drug policy is largely about whether enforcement is too intense, given the very substantial harms associated with enforcement and the limited effectiveness of enforcement in curbing the market in democratic societies. For most of the other markets the policy debate is almost exactly the opposite; why isn’t enforcement tougher? The harms associated with tough enforcement are much less salient than for drug markets. That is not just because so far the enforcement has been slight; it also reflects the lack of a theory of adaptive behavior by market participants that would result in great harms if the enforcement became more stringent. Commercial sex and human smuggling fall in-between the drugs and the other markets, in that there are potentially important harms that result from much tougher enforcement: for example, more violence against sex workers and more risk of death in passage for undocumented immigrants.

## 6. Conclusions and (Tentative) Policy Implications

The markets covered in this Special Feature are heterogenous; they differ in terms of whether they involve products or services, the nature of the players, the geographic range, and their scale. Their commonality is that at least some of their activities either on the demand or on the supply-side have been prohibited or at least heavily restricted. They are all, in other words, the result of policy constructions.

Despite this essential similarity, we have noted considerable variation in procedures and outcomes. Variation attains to the motivation for, and the extent of, the bans; the types of laws applied; the sanctions imposed; the illegal markets’ relation to corresponding legal markets and the intensity of enforcement. The last, in particular, often remains weak and/or inconsistent, because the state authorities lack capacity, need to balance the goal of the reduction of the market or some specific exchanges with the legal interests of other stakeholders, or face difficulties due to the persistent demand, the consensual nature of many transactions, and difficulties of international cooperation.

Notwithstanding the variation and the lack of adequate evaluation for many interventions, some tentative policy conclusions can be reached. The success stories we have reviewed suggest that interventions not focused on law enforcement—such as treatment in the case of drugs—are often effective in reducing the volume of the trades and the associated harms. Some harm reduction initiatives on drug markets have also proven effective, though such initiatives have so far largely targeted only the harms of drug use itself. Conversely, the literature on drug markets shows that tougher enforcement does little to reduce availability, unless it becomes draconian, and then it generates major negative unintended consequences (75).

The experiences of both democratic and authoritarian countries in dealing with illegal drugs suggest that the total suppression of illegal markets, particularly those with a large demand, is neither a realistic nor a defensible goal for polities committed to the respect of human rights. The opposite strategy, legalization, though, also comes with risks. There is growing evidence, for example, that legalization of cannabis production and sales as a weakly regulated commercial industry in many US states is generating considerable use-related harms. Nearly 18 million Americans now report using marijuana daily or near daily—more than the number drinking alcohol that often—and a growing number are enduring addiction, psychosis, and other harms (87, 88).

Nonetheless, the existence of an illegal market for any specific product might still be socially preferable to its absence. Primarily sold legally, tobacco kills more than 7 million people each year (89) and it is estimated that around 100 million people died prematurely because of smoking during the 20th century (90).

The key question then becomes when, where, and how bans on specific products should be enacted in combination with which other instruments. There are large bodies of literatures in law, economics, and criminology on the principles that should guide the criminalization and public regulation of certain kinds of (market) behaviors. While there is no consensus, a dominant view is that a major if not exclusive aim of public control tools—and particularly criminal law—is to prevent potentially harmful activities (91). This goes back to Mill’s (15) harm principle: “the only purpose for which power can be rightfully exercised over any member of a civilized community, against his will, is to prevent harms to others.” Whereas Mill spoke generally of state power, most contemporary legal scholars (92) have restricted the focus to criminal law and advocate for consideration of additional factors (93). Even among the supporters of the harm principle, as Duff and Marshall (94) note, there is no consensus on whether such principle only calls for criminalizing acts that elicit harm or also justifies criminalizing conduct that is not in itself harmful, if such measure can prevent future harm.

Economists usually refer to harm in terms of “negative externalities,” that is, the cost of economic activity on third parties that is not reflected in market prices and is not borne by the decision-maker. Many economists favor taxation (95) or private prosecution (for example via tort or contractual liability) over public controls to deal with externalities. Others have identified the circumstances under which government regulation might be preferred. Building on Coase’s (96) approach to externalities, they support public enforcement when there are high transaction costs between parties and a private solution is likely to fail (97). Although many economists argue that prohibition is likely to create its own harms, even beyond the cost of enforcing the prohibition, Bowles et al. (91) emphasize the comparative advantages of criminal law vis-à-vis civil or administrative law under some circumstances: when the losses are diffuse, and hence civil law does not constitute an alternative, and where they are so large that the risk of insolvency undermines administrative procedures. Children are also preferably subjected to the tighter protection of criminal law because they cannot give full consent; this is why for example child prostitution is prohibited in almost all jurisdictions. In practice, however, civil, administrative, and criminal enforcement tools are mingled in most markets, with the partial exception of drug and, in some countries, sex markets. Even in the case of drugs, and especially cannabis, criminal law is partially giving way to other types of regulation.

Looking ahead, the task thus becomes for both academic research and policymakers to identify and implement the “right” combination of measures—or “meta-strategy,” including the “minimally sufficient punishment” (98)—that allow democratic societies to protect the interests they care for with the minimum level of enforcement-related harm. This meta-strategy is, in other words, inspired by the principle of harm reduction, as it aims at minimizing the harms to the protected interests threatened by the banned activities and the harms generated by policies and interventions. Beyond the adoption of the harm reduction principle in some corners of drug policy, we know only one country, Finland, that has formally adopted harm reduction as a guiding principle for its general criminal policy. Since the 1990s, Finnish criminal policy has been committed to “the minimization of the costs and harmful effects of crime and crime control and the fair distribution of these costs among offender, society, and victim” (99). Even in Finland, though, there has not yet been a formal assessment of how this principle has been translated into practice and to what effects.

Crucially, as many illegal and variable legal markets are cross-border, the search of ideal mix of policies and interventions should take the perspective of an “impartial spectator” (100), considering harms, benefits (that is, the reduction of harms), and costs across countries. The interdiction of, say, cocaine from Colombia to the US is meant to reduce the availability of cocaine in the US, but it may lead to an increase in the export demand for Colombian cocaine, thus worsening Colombia’s drug problem (101). In a cross-national harm reduction perspective, that harm needs to be weighed against the reduction in US drug problems from the modest increase in retail cocaine prices, which might lead to some decline in the prevalence of cocaine use. US and European policy makers have shown minimal interest in considering the consequences of drug policy for mortality, violence and government instability in other countries.

Greenfield and Paoli (102, 103) have developed a “harm assessment framework” to support such analyses, that is, to systematically and empirically assess the harms associated with both banned activities and policy interventions, and to compare the impact, including the unintended consequences and distributional effects, of different types of policies or particular interventions. They speak of a “notional” benefit–cost analysis of the alternatives, because their framework does not require monetization or even quantification of the benefits.

Harm reduction is a staple of drug policy debates, but it has potentially much broader application. Without the label, this kind of reasoning appears in discussions of the regulation of commercial sex. The consequences of policy choices on the spread of disease and violence against women are a core part of debates. It is not merely some measure of the size of the market (number of prostitutes, acts, or customers) that is taken into account. More generally, all efforts at control are likely to have negative unintended consequences as well as (hopefully) desirable intended consequences. Tighter money laundering controls, for example, raise the costs of financial services and increase the number of individuals who are wrongly deprived of access to the banking system. A benefit–cost analysis, encompassing a harm assessment, would take these adverse consequences into account in choosing the most appropriate policy mix and specifically on deciding on the effects of tighter regulation.

We offer these ideas tentatively. Evaluating harm reduction interventions even in the core field of drug use has proven difficult and the studies are often controversial (104). However, harm reduction certainly has the potential to make discussions of regulation of illegal and variably legal markets and enforcement of prohibitions more anchored in a consequentialist, as opposed to moral, frame.

## Data Availability

There are no data underlying this work.
